# An interview with Michael Carmont, section editor for the surgery, traumatology, and rehabilitation section on sports traumatology research: acute, overuse and chronic problems, early return to play and long-term outcomes

**DOI:** 10.1186/2052-1847-5-5

**Published:** 2013-03-28

**Authors:** Michael R Carmont

**Affiliations:** 1 Princess Royal Hospital, Shrewsbury and Telford NHS Trust, Telford, United Kingdom; 2 Northern General Hospital, Sheffield Teaching Hospitals NHS Foundation Trust, Sheffield, United Kingdom

## What does the sports traumatology field involve?

The discipline of sports traumatology broadly encompasses injuries sustained at all levels and in all aspects of a person’s life. Each level of training has different motivations and goals. For example, adolescent athletes have aspirations for the future but may not yet have reached adult development. However, elite athletes compete either at the top of their game or at a professional level, whereas recreational athletes participate and compete for personal ambition, the challenge involved, and the associated health benefits. All athletes are keen to have their injuries managed as quickly and effectively as they can be so that they may return to sports as soon as possible. However, sports surgeons need to be mindful of making decisions for the patient’s long term good rather than a short term, rapid return to play, fix. Primum-non-nocere.

## When and where do injuries normally occur?

Injuries frequently occur during participation in sport at the limit of the athlete’s ability, either in terms of training frequency and intensity, or the capacity of their biological tissues to sustain high load and competition performance.

The injuries most frequently sustained tend to involve tissues that are known to have reduced healing potential and can lead to instability or dysfunction. These tissues typically involve articular cartilage, meniscus, ligament and tendon. Jack Hughston, one of the fore fathers of sports surgery, has advised “Whenever you are having your anatomy sessions, pay particular attention because orthopedics is all about anatomy…plus a little bit of common sense.”

## We appreciate that you have a specialist interest in treating knee injuries; what are the main developments in this area and where are we today?

The cycle of sports treatment means that primary repair is performed and is often followed by the adoption of new techniques and technology, which can then be evaluated against the success of the primary repair. Rupture of the anterior cruciate ligament leads to knee laxity and subjective knee instability may prevent return to play.

Originally, early primary repair of internal derangement was considered to attempt to restore the original anatomy [1]. Once the process of anterolateral rotational instability was understood, lateral tenodesis used biomechanical principles to stabilize the joint [2]. Internal reconstruction became more favorable but questions continue regarding graft selection and precise, more anatomical, tunnel positioning. Should we be using double bundle reconstruction [3,4]? Surgeons are failing to show a convincing difference between techniques and consideration is being given to returning to augment reconstruction with lateral tenodesis [5]. The pitfalls of graft harvest are also being appreciated: bone patella tendon bone harvest can lead to anterior knee pain and pain on kneeling [6], and hamstring harvest can reduce deep flexion torque of the knee [7]. The confidence in graft healing has lead to less tissue harvest and resultant morbidity [8]. As a result, once again surgeons are revisiting primary repair, with healing response [9] and augmentation [10].

## What are the main problems encountered during knee surgery on unstable knees?

In the knee, meniscal tears can be debilitating but are easily treated by arthroscopic partial meniscectomy although there are costs involved [11]. For example, meniscal repair requires technical skill and may protect against the development of osteoarthritis, but prolongs surgical and rehabilitation time [12].

Articular cartilage defects provide a perplexing problem for surgeons. The structure itself is aneural, avascular and alymphatic and so the question is raised as to why these are painful? Tissue regeneration covers the exposed bone, but restoration of hyaline cartilage is difficult compared to the formation of fibro-cartilage with reduced biomechanical properties. The best results from the process of micro-fracture are produced with significant rehabilitation commitment [9] and long-term outcome has been proven [13], meaning that these techniques have now become the baseline by which new methods are compared [14]. However, the results of alternative procedures may be tarnished by previous surgery and so direct comparison is difficult.

## Are there any other particular injuries that are especially difficult to manage?

Yes. Tendinopathy in particular occurs with symptoms of pain, thickening and dysfunction [15] and yet the cause is not understood. Neo-vascularisation has been postulated, treated by injections, sclerosis, coblation and stripping [16-18]. However, given the effectiveness of these treatments we are beginning to rethink again [19]. For example, in the Achilles tendon, attention has now progressed to the adjacent plantaris [20]. Once again treatment options must be compared with therapy to load the tendon to reduce symptoms [21].

## Where are we with helping athletes to effectively regain their full potential after injury?

Athletes strive to be the best they can be and are willing to adopt any practice that will allow them to gain advantage over a rival and this also applies to recovery from injury.

The use of modalities to promote healing has been promoted over the last few years. The introduction of particular concentrations of growth factors to enhance healing and accelerate recovery has now been adopted in sports medicine. To date, few randomized controlled studies have shown improvements in clinical outcome [22] despite convincing evidence in basic science [23], but research has been complicated by variations in pathology [24], preparation [25,26], and recruitment. Which athletes would wish to be in the control arm of a treatment that has little disadvantages and risks and yet may confer a benefit in outcome? Perhaps the perceived benefits of this procedure has meant that desire to return to play has prevented the true effectiveness of this technique being established.

## As a clinician in the field, what do you think is the most important aspect to consider when treating athletes, both now and in the future?

In all areas of sports science we have a duty to our athletes, patients, and future patients to evaluate new techniques and compare these with established methods for both early and long-term outcomes. Science needs to advance but we need to respect the activity and experience of our wise predecessors.

## Authors’ information

Michael Carmont is Consultant Orthopaedic Surgeon working at the Princess Royal Hospital, Telford and Northern General Hospital, Sheffield. He is interested in sports injuries of the knee, foot and ankle, and has a specialist interest in anterior cruciate ligament reconstruction and the management of Achilles tendon rupture. He is the Section Editor for the Surgery, traumatology, and rehabilitation section of *BMC Sports Science, Medicine and Rehabilitation*. In this interview we find out a little more about the key issues in this field of research.

## Pre-publication history

The pre-publication history for this paper can be accessed here:

http://www.biomedcentral.com/2052-1847/5/5/prepub
